# A description of variant transthyretin amyloidosis (ATTRv) stage 1 patients and asymptomatic carriers in Spain: the EMPATIa study

**DOI:** 10.1186/s13023-024-03304-9

**Published:** 2024-09-06

**Authors:** Lucía Galán Dávila, Fernando Martinez Valle, Juan Buades Reinés, Juan Gonzalez-Moreno, Inés Losada López, Teresa Sevilla, Francisco Muñoz Beamud, José Eulalio Bárcena Llona, Manuel Romero Acebal, Francesca Setaro, Diana Primiano, Patricia Tarilonte

**Affiliations:** 1 Neurology Department, Hospital Clínico San Carlos, IdISSC, Madrid, Spain; 2 Internal Medicine Department, Hospital Universitario Vall d’Hebron, Barcelona, Spain; 3 Internal Medicine Department, Hospital Universitario Son Llàtzer, Palma, Spain; 4 Balearic Research Group in Genetic Cardiopathies, Sudden Death and TTR Amyloidosis, Instituto de Investigación Sanitaria de las Islas Baleares (IdISBa), Palma, Spain; 5 Neurology Department, Hospital Universitari i Politècnic La Fe & IIS La Fe, Valencia, Spain; 6 Centro de Investigación Biomédica en Red de Enfermedades Raras (CIBERER), Madrid, Spain; 7 Internal Medicine Department, Hospital Universitario Juan Ramón Jiménez, Huelva, Spain; 8 Neurology Department, Hospital Universitario Cruces, Barakaldo, Bizkaia Spain; 9 Neurology Department, Hospital Universitario Virgen de la Victoria, Málaga, Spain; 10 Pfizer S.L.U., Madrid, Spain; 11 Neurology Department, Universitat de València, Valencia, Spain

**Keywords:** Amyloid, ATTRv mutation, Asymptomatic carriers, Endemic, ATTRv red flags, SUDOSCAN

## Abstract

**Background:**

Variant transthyretin amyloidosis (ATTRv) is a rare multisystemic disorder caused by mutations in the transthyretin (TTR) gene. The aim of the present work was to describe the clinical profile of asymptomatic carriers (AC) and *Coutinho* stage 1 ATTRv patients in Spain.

**Methods:**

National, multicentre, cross-sectional study that included 86 AC and 19 patients diagnosed in the previous 12 months to enrolment. Clinical and demographical data, *TTR* gene mutations, red flags anamnesis, neurological and cardiological assessments were collected.

**Results:**

The mean age of patients was 56.8 years at onset and 58.6 years at diagnosis; 53% of patients and 51% of AC were from non-endemic areas. Val50Met was the most frequent mutation in both groups. Neuropathy impairment score data (mean 17.7 ± 20.5) and small-fibre function in lower limbs assessed with SUDOSCAN revealed that patients were diagnosed at early stages of neurological impairment. Peripheral polyneuropathy (84.2%), autonomic neuropathy (73.7%), cardiac (63.2%) and gastrointestinal (47.4%) alterations were the most common symptoms in patients. Autonomic neuropathy, gastrointestinal impairment, carpal tunnel syndrome, cardiac and ocular alterations were potentially related to ATTRv in the AC group.

**Conclusions:**

The EMPATIa study provides a detailed description of AC and *Coutinho* stage 1 ATTRv patients across Spain, confirming the multisystemic clinical profile of the disease. This study reveals a diagnosis delay around 1.8 years, highlighting the importance of a profound disease awareness to reach a diagnose in earlier stages of neurological impairment.

**Supplementary Information:**

The online version contains supplementary material available at 10.1186/s13023-024-03304-9.

## Background

Variant transthyretin amyloidosis (ATTRv) is a rare, progressive, clinically heterogenous and life-threatening disease caused by extracellular deposition of amyloid fibrils formed of mutant transthyretin (TTR) [1–3]. It is an autosomal-dominant disorder with an incomplete penetrance and an age of onset ranging from the second to the ninth decade of life [4].

TTR is a transport protein mainly synthesized in the liver [5]. Pathogenic mutations increase the intrinsic tendency of the TTR tetramer to misfold into dimers and monomers, which aggregate into oligomers and insoluble amyloid fibrils. These amyloid fibrils accumulate in different organs and tissues, particularly in the peripheral and autonomic nervous systems and in the heart [1], but also in the eyes or kidneys [4], resulting in systemic dysfunction [6].

More than 150 mutations in the *TTR* gene have been identified so far [1, 7, 8]. Although certain variants are typically associated with polyneuropathy (PN) (i.e., Val50Met) and others typically result in cardiomyopathy (i.e., Val142Ile), the phenotypic pattern may overlap [1].

Diagnosis of the disease is a challenge due to the lack of specific symptoms and considerable variability among patients. A systematic and multidisciplinary follow-up of asymptomatic carriers (AC) is imperative to enable detection of the earliest symptoms of the disease, and is the key to timely treatment to delay disease progression [4, 9]. A set of signs and symptoms, commonly known as red flags, have been drawn up to assist in the early detection of the disease [10].

ATTRv is a low-prevalence disease; however, prevalence in endemic areas such as northern Portugal, northern Sweden, and specific regions in Japan, Cyprus, Bulgaria, and Brazil (endemic areas) is higher. In Spain, the endemic regions with higher prevalence are Mallorca (Balearic Islands) and Valverde del Camino (Huelva) [11].

Given the scarcity of evidence and data on ATTRv patients and carriers in Spain [11–14], this study was performed to describe the clinical profile of AC of pathogenic *TTR* gene mutations and patients with *Coutinho* stage 1 ATTRv with polyneuropathy (PN), diagnosed in the last 12 months in Spain.

## Results

### Demographics

A total of 112 subjects enrolled in the EMPATIa study, 105 were eligible, 19 of whom were *Coutinho* stage 1 polyneuropathy ATTRv patients and 86 AC of a pathogenic mutation in the *TTR* gene (Supplementary Fig. S1). Demographic and clinical characteristics of both groups are shown in Table 1. More than half of all patients and AC were recruited from non-endemic sites and were men. Patients were significantly older than AC. The median age of patients from non-endemic areas was higher compared with patients from endemic areas, but this difference was not statistically significant (67.5 vs. 60.5, *p* = 0.253). The mean age of patients at symptom onset was lower than the mean age at diagnosis, revealing a diagnosis delay of 1.8 years. The mean age of patients at symptom onset from endemic areas was 52.6 years and from non-endemic areas it was 59.7 years. The mean age at diagnosis from endemic areas was 55.8 years, and in patients from non-endemic areas it was 61.1 years. Patients had a family history of ATTRv in 63.2% of cases, whereas the remaining 36.8% were the first to be diagnosed in their families. The median age and IQR of index patients, defined as the first patient in a family who has a debut with the disease at diagnosis, was 60.0 (50.0–70.0) years, with son/daughter being the most frequent AC relationship to the index patient (Table 1). Index patients from non-endemic area were significantly older than those from endemic area (median age 64.0 vs. 50.0 years, *p* = 0.007).

**Table 1:** Clinical and sociodemographic characteristics

	ATTRv patients N = 19	Asymptomatic carriers N = 86	*p* value
Age (years), median (Q25–Q75)	67.0 (37.1–77.2)	44.7 (18.1–81.8)	0.001
Men, n (%)	11 (57.9)	48 (56.5)	NS
Non-endemic site, n (%)	10 (52.6)	44 (51.2)	NS
Residence*, n (%)			
Balearic Islands (end. area)	7 (36.8)	26 (30.2)	
Madrid	3 (15.8)	10 (11.6)	
Huelva (end. area)	2 (10.5)	13 (15.1)	
Barcelona	2 (10.5)	12 (14.0)	
Valencia	2 (10.5)	9 (10.5)	
Others	3 (15.8)	16 (18.6)	
Place of family origin*, n (%)			
Balearic Islands	7 (36.8)	28 (32.6)	
Huelva	2 (10.5)	14 (16.3)	
Valencia	2 (10.5)	8 (9.3)	
Madrid	2 (10.5)	7 (8.1)	
Castellon	1 (5.3)	5 (5.8)	
Others	5 (26.3)	24 (27.9)	
Age at symptom onset, years, median (Q25–Q75)	65.0 (43.0–69.0)		
Age at symptom onset, years, mean (SD)	56.8 (14.5)		
Age at diagnosis, years, median (Q25–Q75)	66.0 (46.0–71.0)		
Age at diagnosis, years, mean (SD)	58.6 (13.9)		
Family history, n (%)	12 (63.2)	86 (100)	
Relationship to index patient, n (%)			
Son/daughter		31 (36.0)	
Nephew/niece		11 (12.8)	
Aunt/uncle		2 (2.3)	
Father/mother		12 (14.0)	
Sister/brother		9 (10.5)	
Cousin		9 (10.5)	
Grandchild		8 (9.3)	
Other		4 (4.7)	
Index patient’s age at diagnosis, median (Q25–Q75)		60.0 (50.0–70.0)	
Index patient’s age at diagnosis, mean (SD)		58.2 (14.3)	
Modified BMI, kg/m^2^ × g/dl, mean (SD)	1068.7 (155.7)	1157.5 (208.6)	NS
HBP	6 (31.6%)	15 (17.4%)	NS

*BMI* body mass index, *end* endemic, *HBP* high blood pressure, *NS* not significant, *SD* standard deviation

*The 5 most relevant

### Genotype

As shown in Table 2, Val50Met was the most common mutation in both patients (84.2%) and AC (77.9%). It was the sole mutation identified in endemic areas, but was only identified in 70.0% of patients and 56.8% of AC in non-endemic areas. Both early- (< 50 years) and late-onset (≥ 50 years) Val50Met patients were identified in endemic and non-endemic areas with a proportion 1:2 and 1:6 respectively. In patients, other mutations identified were Ser97Tyr and Glu109Lys. In AC, other mutations identified were Ser97Tyr, Val142Ile, Asp38Asn and Thr80Ala. The genetic study involved complete sequencing of the *TTR* gene in 10 (55.6%) patients and 21 (28.8%) AC, exon 2 analysis in 4 (22.2%) patients and 38 (52.1%) AC, exon 3 analysis in 3 (16.7%) patients and 14 (19.2%) AC, and restriction fragment length polymorphism (RFLP) in 1 (5.6%) patient. All subjects analysed were heterozygous, except for 1 AC identified as compound heterozygous. Polymorphism analysis was performed in 15 patients and 70 AC, showing the presence of the Gly26Ser polymorphism in 5 (33.3%) patients and 18 (25.7%) AC.

**Table 2:** Genotype distribution

	ATTRv patients N = 19	Asymptomatic carriers N = 86	*p* value
TTR mutations, n (%)			NS
Val50Met	16 (84.2)	67 (77.9)	
Val142Ile	0 (0.0)	7 (8.1)	
Ser97Tyr	1 (5.3)	9 (10.5)	
Glu109Lys	2 (10.5)	0 (0.0)	
Asp38Asn	0 (0.0)	2 (2.3)	
Thr80Ala	0 (0.00)	1 (1.2)	
Heterozygote	19 (100.0)	83 (98.8)	NS
Compound heterozygote	0	1 (1.2)	

*Ala* alanine, *Asn* asparagine, *Asp* aspartic acid, *Glu* glutamic acid, *Ile* isoleucine, *Met* methionine, *NS* not significant, *Ser* serine, *Thr* threonine, *Tyr* tyrosine, *Val* valine

### Red flags in patients and asymptomatic carriers

A review of ATTRv red flags showed that among the autonomic neuropathy manifestations (73.7%), sweating abnormalities were the most common. Cardiovascular manifestations were the second red flag more prevalent in ATTRv patients (63.2%), where cardiomyopathy was the most present demonstration (Table 3). Incidence of gastrointestinal symptoms was in 47.4% of patients, where diarrhoea and early satiety were the most frequent signs. Carpal tunnel syndrome was found in 31.6% of patients. Peripheral sensory-motor neuropathy, a characteristic condition of overt disease, was only found in patients (84.2%). Nevertheless, 3 patients—2 from non-endemic area and 1 from endemic area—presented autonomic neuropathy without peripheral sensory-motor polyneuropathy. Ocular manifestations and CNS alterations (including mild manifestations such as headaches) were also encountered in 21.1% and 15.8% of patients, respectively (Table 3).

**Table 3:** ATTRv red flags

	ATTRv patients N = 19	Asymptomatic carriers N = 86	*p* value
Peripheral sensory-motor neuropathy, n (%)	16 (84.2)	0 (0.0)	< 0.001
Autonomic neuropathy, n (%)*	14 (73.7)	14 (16.3)	< 0.001
Orthostatic hypotension	3 (21.4)	4 (28.6)	
Recurrent urinary tract infections due to urinary retention	1 (7.1)	1 (7.1)	
Sexual dysfunction	6 (42.9)	2 (14.3)	
Sweating abnormalities	6 (42.9)	6 (42.9)	
Cardiovascular manifestations, n (%)*	12 (63.2)	10 (11.6)	< 0.001
Conduction block	2 (16.7)	1 (10.0)	
Cardiomyopathy	9 (75.0)	5 (50.0)	
Arrhythmia	1 (8.3)	1 (10.0)	
Gastrointestinal manifestations, n (%)*	9 (47.4)	13 (15.1)	0.004
Nausea and vomiting	1 (11.1)	(7.7)	
Early satiety	3 (33.3)	(0.0)	
Diarrhoea	6 (66.7)	7 (53.8)	
Alternating episodes of diarrhoea and constipation	1 (11.1)	0 (0.0)	
Unintentional weight loss	3 (33.3)	0 (0.0)	
Carpal tunnel syndrome, n (%)	6 (31.6)	10 (11.6)	0.040
Ocular manifestations, n (%)	4 (21.1)	5 (5.8)	NS
Central nervous system manifestations, n (%)*	3 (15.8)	6 (7.0)	NS
Transient ischemic attack-like episodes	1 (33.3)	1 (16.7)	
Nephropathy, n (%)	1 (5.3)	0 (0.0)	NS
Proteinuria	1 (100.0)	0 (0.0)	

*Percentages in each type of manifestation attributed to a particular red flag group was calculated from the n in that group, not from N. Since it is a multiple response, the sum of the percentages can be greater than 100%

Signs of red flags compatible with ATTRv symptoms were identified in the AC group (Table 3). The most frequent alterations in AC were autonomic neuropathy symptoms—mainly sweating abnormalities, sexual dysfunction and orthostatic hypotension—in 14 (16.3%), gastrointestinal disturbances—mainly diarrhoea—and cardiac alterations in 10 (11.6%), of whom 5 (50.0%) presented cardiomyopathy. Carpal tunnel syndrome was observed in 10 (11.6%) AC; 6 (7.0%) presented CNS symptoms, and 5 (5.8%) ocular manifestations.

### Neurological test evaluation and quality of life (QoL)

Neurological assessment of small-fibre performed using the SUDOSCAN showed no-pathological mean ESC scores in hands, whereas mean lower limb scores were pathological in patients’ group (Table 4). Patients in non-endemic areas presented significantly lower ESC scores in hands compared with endemic areas (60.0 µS vs. 74.7 µS; *p* = 0.034), although no differences were found in ESC scores in the feet. AC from endemic areas presented significantly lower ESC scores in comparison with AC from non-endemic areas (mean 74.5 µS vs. 80.1 µS, *p* = 0.033).

**Table 4:** Cardiological, neurological and biochemical assessment

	N	ATTRv patients	N	Asymptomatic carriers	*p* value
Cardiological assessment					
Electrocardiogram					
QRS, ms, mean (SD)	10	0.10 (0.02)	53	0.10 (0.07)	NS
QT, ms, mean (SD)	13	347.5 (33.1)	54	367.9 (56.3)	NS
R–R interval, ms, mean (SD)	10	658.7 (196.2)	52	812.0 (311.4)	0.011
CVR–R,%, mean (SD)	6	4.2 (2.4)	37	12.7 (11.6)	0.003
Atrial fibrillation, n (%)	15	0 (0.0)	64	0 (0.0)	NS
LV hypertrophy signs, n (%)	15	3 (20)	64	2 (3.1)	0.045
Atrioventricular block, n (%)	15	2 (13.3)	64	1 (1.6)	NS
Branch block, n (%)	15	1 (6.7)	64	2 (3.1)	NS
Pseudoinfarction pattern, n (%)	15	3 (20.0)	64	4 (6.3)	NS
Echocardiogram					
LV ejection fraction, %, mean (SD)	8	60.8 (10.0)	30	64.7 (7.1)	NS
IVS thickness, mm, mean (SD)	11	15.2 (3.9)	30	10.0 (1.8)	< 0.001
Tilt-Test Positive, n (%)	7	1 (14.3)	14	1 (7.1)	NS
Neurological assessment					
NIS, mean (SD)	18	17.7 (20.5)	67	0.8 (2.1)	< 0.001
NORFOLK QoL-DN, mean (SD)	17	46.6 (28.9)	68	6.9 (10.4)	< 0.001
CCI, mean (SD)	12	2.7 (2.4)	57	0.4 (0.8)	< 0.001
ESC Sudoscan, µS, mean (SD)					
Lower limbs	17	54.7 (28.7)	80	77.1 (11.6)	0.005
Upper limbs	16	68.3 (14.5)	80	72.4 (12.8)	NS
Biochemical data					
ProBNP, pg/mL, mean (SD)	5	1269.8 (1109.3)	16	178.9 (476.0)	0.002
NT-proBNP, pg/mL, mean (SD)	12	427.3 (560.1)	41	64.1 (72.6)	0.002
Troponin T, µg/L, mean (SD)	2	25.8 (24.7)	8	5.1 (6.1)	NS
Troponin I, µg/L, mean (SD)	2	35.5 (0.7)	24	8.1 (39.0)	0.029
Pre-albumin (transthyretin), mg/dL, mean (SD)	11	24.7 (8.1)	45	23.4 (6.6)	NS
Microalbuminuria, mg/dL, mean (SD)	6	7.2 (4.0)	41	12.8 (12.6)	NS
Proteinuria, (g/24), mean (SD)	5	6.8 (6.3)	29	22.0 (38.1)	NS

*CCI* Charlson comorbidity index, *CVR–R* coefficient of variation of R–R intervals, *ESC* electrochemical skin conductance, *IVST* interventricular septal thickness, *LV* left ventricular, *NIS* neuropathy impairment score, *NORFOLK QoL-DN* Norfolk quality of life-diabetic neuropathy questionnaire, *NT-proBNP* N-terminal pro-brain natriuretic peptide, *ProBNP* pro-brain natriuretic peptide, *R–R interval* the time elapsed between two successive R-waves, *QRS* Q, R and S waves, *QT* Q–T interval, *SD* standard deviation

Predictably, NIS scores in patients were significantly higher than in AC (Table 4), and scores in AC from non-endemic areas were significantly higher than those from endemic areas (mean 1.1 vs. 0.3, respectively, *p* = 0.023). Consistent with these results, the Norfolk QoL-DN questionnaire scores showed that quality of life in stage 1 patients was worse than in AC, and Charlson comorbidity index (CCI) score was categorized as mild in patients (Table 4). In line with these data, only 11.6% of AC received symptomatic treatment versus 63.2% of patients (*p* < 0.001).

### Cardiological assessment

Echocardiography results were shown in Table 4. Median thickness of the interventricular septum was significantly higher in patients than AC, being > 14 mm in 54.5% (6/11) of patients and in 3.3% (1/30) of AC, between 12 and 14 mm in 36.4% (4/11) of patients and in 16.7% (5/30) of AC, and < 12 mm in 9.1% (1/11) of patients and in 80.0% (24/30) of AC. No differences between groups on left ventricular ejection fraction (LVEF) was observed.

An analysis of ECG results showed that none of the patients or AC exhibited atrial fibrillation (Table 4). ECG alterations were more common in patients compared with AC statistically significant differences were observed in left ventricular hypertrophy signs and R–R interval variation. Additional data on the cardiological assessment are shown in Table 4.

### Biochemical parameters

Mean NT-proBNP levels were significantly higher in patients, while in AC statistically significant differences in mean NT-proBNP levels were observed between endemic and non-endemic areas (81.5 ± 86.1 pg/mL vs. 37.0 ± 30.2 pg/mL, *p* = 0.016). Troponin I levels were also significantly higher in patients than in AC (*p* < 0.05). No significant differences were observed in pre-albumin/TTR, troponin T, retinol-binding protein 4 (RBP4), microalbuminuria, or estimated glomerular filtration rate (eGFR) between patients and AC (Table 4). Nevertheless, higher levels of vitamin A and RBP4 were detected in AC from non-endemic areas compared with endemic areas (*p* = 0.001), while higher levels of microalbuminuria and proteinuria were found in AC from endemic areas compared with non-endemic areas (*p* = 0.001 and *p* < 0.001, respectively).

## Discussion

EMPATIa is a descriptive study of a representative population of ATTRv-PN amyloidosis stage 1 patients and AC from endemic and non-endemic areas in Spain. Prior to this study, most of the evidence available derived from studies performed in endemic areas such as Mallorca and Valverde del Camino in Huelva, and little information was available from other regions of Spain. This study, carried out in experienced hospitals from both endemic and non-endemic areas, contributes important information and broadens the understanding of the disease pattern in Spain [11, 12, 15].

Approximately half of the study population was from and lived in non-endemic regions. Similar observations were reported in the THAOS (Transthyretin Amyloidosis Outcome Survey) registry [12], which showed that the disease was widely distributed across Spain, and that clinicians from non-endemic areas need to be aware of its presence [12].

Identification of ATTRv-PN can be challenging, particularly in non-endemic regions. Its systemic and heterogeneous clinical profile, low prevalence, and the belief that ATTRv affects younger people and/or those with ATTRv-diagnosed relatives, make correct and early diagnosis a difficult task [16, 17]. EMPATIa study results showed that ATTRv-PN stage 1 patients have more neurologic alterations at lower than upper limbs, pointing to an early diagnosis. The mean diagnostic delay (the difference between the mean age of the patient at diagnosis and the mean age at first onset of symptoms) reported by experienced hospitals participating in the EMPATIa study was around 1.8 years (58.6 vs. 56.8). This delay was shorter than the 3–4-year delay in non-endemic regions reported in earlier studies, highlighting that greater awareness of the disease may reduce diagnostic delays [2, 18].

ATTRv-PN was more prevalent in male patients, with a median age of 67 years, with Val50Met being the most common mutation. The mean age of patients from non-endemic areas and their mean age at onset of symptoms are higher compared with patients from endemic areas, showing that the late-onset profile is common in non-endemic regions in Spain. However, as previously described, late-onset patients were identified in both endemic and non-endemic regions [12, 17]. While peripheral sensory-motor neuropathy, autonomic neuropathy, cardiac alterations and gastrointestinal impairment were the most common ATTRv-related symptoms in patients, autonomic neuropathy and cardiac alterations were more common in endemic areas, and CNS and ocular manifestations were only found in these latter patients. A recent analysis from THAOS Spanish data found that patients mainly presented a neurologic or mixed phenotype with cardiac and gastrointestinal involvement [12]. This is evidence of the systemic nature of ATTRv, and shows the need for a multidisciplinary approach focused not only on neurological assessment but also on routine cardiac, ocular, and renal monitoring.

The EMPATIa study characterises genotype distribution in Spain, showing that Val50Met was the most common mutation in both groups, and the only one found in endemic areas. In non-endemic areas, 70% of patients and 56.8% of AC had the Val50Met mutation. For patients, Ser97Tyr (10%) and Glu109Lys (20%) were the other mutations detected; Ser97Tyr (20.5%) and Val142Ile (15.9%) were the most common non-Val50Met mutations encountered for AC.

At the design stage of the study, in 2017, the criterion chosen to discriminate between AC and patients was the presence of PN, mostly diagnosed on the basis of large fibre alteration in the electromyogram. However, clinical decision was the final criterion patients’ diagnosis, and hence 3 individuals—2 from non-endemic areas and 1 from an endemic area—who presented autonomic dysfunction without sensory-motor polyneuropathy were also classified as patients. As expected, the presence of possible signs compatible with ATTRv amyloidosis red flags were found in the clinical records of this population of AC: 16.3% presented potential signs of autonomic neuropathy, such as sweating abnormalities or orthostatic hypotension; 15.1% complained of gastrointestinal disturbances, mostly diarrhoea (53.8%) and nausea (7.7%); 11.6% presented carpal tunnel syndrome, and 5.8% had ocular manifestations, mostly vitreous opacities (40%). Furthermore, 11.0% of AC showed cardiological alterations: conduction disorders (10.0%), cardiomyopathy (50.0%), and arrhythmia (10.0%). Regarding the five AC with cardiomyopathy, the study design does not allow to determine which of them could be considered patient under the latest diagnostic criteria [9]. Therefore, following international consensus for early diagnosis in ATTR [9], up to 16.3% of AC presenting autonomic neuropathy could probably have been classed as patients. This emphasizes the need for a more thorough review of the clinical history and a multidisciplinary approach, even in apparently healthy carriers.

For subjects with a known family history of ATTRv, any onset of length-dependent axonal polyneuropathy predominantly affecting temperature and pain sensation, autonomic dysfunction or specific cardiac alterations indicates the need to assess a potential organ involvement [2]. Early markers of the onset of ATTRv are needed to identify patients in whom other systems or organs could be involved, such as skin [19], heart [20, 21], sweat glands [22], or cornea [23]. Timely diagnosis of ATTRv is critical for appropriate treatment and optimal outcomes [4].

This study has some limitations. First, as previously mentioned, the criterion used to discriminate between AC and patients at the time of study design (2017) was the presence of PN; however, given the current understanding of ATTRv, some of the subjects considered AC at that time might have been more correctly classed as patients. In this study no data about specific disease-modifying treatment was collected and hence results could not be reviewed according to this variable. This study has in addition the limitations inherent to a cross-sectional observational study also. Due to the small sample size, particularly in the case of patients, any significant findings in this study will need to be confirmed in a larger population in order to minimize spurious associations. A larger sample size would have allowed to perform other analyses and find more robust associations. However, sample size is a common limitation in the study of rare diseases.

## Conclusions

The EMPATIa study provides a description of representative Spanish populations of *Coutinho* stage 1 ATTRv patients and AC diagnosed in the 12 months prior to recruitment, between 2017 and 2020. These results show that the disease is widely distributed across Spain, where the most common mutation is the Val50Met and that patients predominantly exhibit a mixed phenotype, where cardiac and gastrointestinal alterations are the most common extra-neurological manifestations. Mean age of patients included in the study from non-endemic areas, and their mean age at disease onset are higher when compared with those from endemic areas, showing that late-onset profile is common in non-endemic regions.

Patients included in this study were diagnosed at early stages of neurological impairment, confirming the importance of diagnosing at an early stage, before they present large fibre involvement, according to the latest international consensus [2, 9]. Results showed that a 36.8% of ATTRv-PN patients were the first to be diagnosed in their families, pointing out the importance to disseminate data regarding ATTRv red-flags across non-experienced healthcare professionals. In conclusion, this study shows that a high level of disease awareness and a multidisciplinary approach for the follow-up of both ATTRv-PN patients and asymptomatic carriers allow a reduction of the diagnostic delay, then reshaping patient’s clinical peregrination.

## Methods

### Study design and population

EMPATIa is a national, multicentre, cross-sectional, non-interventional study performed in 7 hospitals in Spain—2 in endemic areas (Huelva and Mallorca) and 5 in non-endemic areas (Madrid, Barcelona, Vizcaya, Málaga and Valencia). The *primary endpoint* was to analyse demographic, laboratory and clinical variables to determine the profile of AC of pathogenic mutations in the *TTR* gene and that of patients with *Coutinho* stage 1 ATTRv diagnosed in Spain in the previous year. The *secondary endpoints* were to determine AC and *Coutinho* stage 1 patient populations by site or region, and the differences between endemic and non-endemic areas in the Spanish population. Both patients and AC who gave their informed consent were included in the study, which was approved by the regional Ethics Committee and performed in accordance with the Declaration of Helsinki and applicable local laws and regulations. Inclusion criteria for AC of pathogenic TTR mutations were age ≥ 18 years and confirmation of pathogenic TTR mutation. Inclusion criteria for patients were age ≥ 18 years, confirmed diagnosis of ATTRv with *Coutinho* stage 1-PN in the 12 months prior to recruitment.

Patients and AC with comorbidities such as diabetes *mellitus*, *Coutinho* stage 2/3 PN ATTRv, monoclonal gammopathy of undetermined significance, other concomitant diseases that the investigators believe could mimic ATTRv, pregnant or breastfeeding women, and those who refused to give their informed consent were excluded. In 2017, when the study was designed, most hospitals identified the disease as familial amyloidotic polyneuropathy (FAP), and the basis for discriminating between AC and patient was the presence of large-fibre involvement in the electromyogram. Therefore, the criterion used to discriminate between AC and patients in this study was the presence of PN, confirmed by electromyogram, regardless of the presence of dysautonomia or extra-neurological involvement.

### ATTRv amyloidosis red flags

The anamnesis of ATTRv red flags [10] was collected from the clinical history and categorized as follows: autonomic and peripheral sensory-motor neuropathies, central nervous system (CNS) manifestations, ocular, kidney, gastrointestinal and cardiac alterations, and carpal tunnel syndrome. Autonomic neuropathy was defined as the presence of any of the following symptoms: orthostatic hypotension, recurrent urinary tract infections due to urinary retention, sexual dysfunction, or sweating disturbances. These symptoms were collected from both patients and AC, and when found in AC they were considered to be clinical alterations compatible with ATTRv.

### Sudomotor function

The SUDOSCAN (Impeto Medical; Paris, France) is a simple, non-invasive device that indirectly assesses sudomotor function by measuring electrochemical skin conductance (ESC) [24] in the hands and feet. ESC is considered normal if it exceeds 70 and 60 μS in the feet and hands, respectively [25].

### Clinical outcomes

Data from AC and patients were collected from clinical records and individual patient interviews conducted during a single study visit that took place between June 2018 and July 2020.

In order to describe the demographic and clinical characteristics of Spanish AC and patients, the investigators collected demographic and clinical data including age, sex, weight, height, modified body mass index (mBMI), mutation (homozygosis/heterozygosis, polymorphisms, genotyping technique), employment status, place of residence and province of family origin, comorbidities, and ATTRv-specific symptoms (red flags) from both patients and AC. In the case of patients, age at symptom onset and date of diagnosis were collected. In the case of AC, relationship with index patient and age of index patient at disease onset were collected.

The following data were collected from health records: (1) sensory-motor and autonomic neurological impairment determined by electromyography along with autonomic nervous system studies; (2) cardiovascular autonomic dysfunction determined using a Tilt test and the R–R interval (time elapsed between two successive R waves of the QRS signal on the electrocardiogram); (3) sudomotor function and ESC assessed on the SUDOSCAN; (4) ATTRv-related neurological status determined by the neuropathy impairment score (NIS) [26]; (5) quality of life (QoL) measured by the Norfolk Quality of Life Questionnaire for Diabetic Neuropathy (Norfolk QoL-DN) [27–29]; (6) Charlson Comorbidity Index (CCI) score; (7) cardiac function determined by electrocardiogram (ECG) and echocardiogram (ECHO) studies; (8) laboratory data, including serum levels of biomarkers such as the N-terminal prohormone of brain natriuretic peptide (NT-proBNP), troponin T and I, albumin and transthyretin levels, as well as evidence of microalbuminuria and proteinuria. The NIS is a composite score derived from the neurological evaluation of sensory function, reflexes, and muscle weakness. Muscle weakness is scored from 0 to 192, reflexes are scored from 0 to 20, and sensation (upper and lower limbs) is scored from 0 to 32, giving a total score of between 0 to 244, with higher scores indicating greater impairment. The Norfolk QoL-DN assesses how neuropathy affects a patient’s life, with higher scores indicating lower quality of life.

### Statistical analysis

Baseline demographics and clinical variables were summarized as numbers and percentages of patients in the case of categorical variables, and medians and interquartile ranges or means and standard deviations (SD) in the case of continuous variables or frequencies as appropriate. Categorical variables were compared using Chi-squared (χ^2^) tests and Fisher tests, and continuous variables were compared using the two-tailed Student’s t test and Mann–Whitney-U test. Tests were two-tailed with a significance level of 5%.

## Supplementary Information

Supplementary Material 1.

## Data Availability

The datasets used during the current study are not openly available due to reasons of sensitivity and are available from the corresponding author on reasonable request. Data are located in controlled access data storage at Pfizer SLU (Spain).

## Abbreviations

AC: Asymptomatic carrier
ATTRv: Variant transthyretin amyloidosis
BMI: Body mass index
CCI: Charlson comorbidity index
CNS: Central nervous system
CVR–R: Coefficient of variation of R–R intervals
ESC: Electrochemical skin conductance
ECG: Electrocardiogram
ECHO: Echocardiogram
eGFR: Estimated glomerular filtration rate
end: Endemic
FAP: Familial amyloidotic polyneuropathy
HBP: High blood pressure
IVST: Interventricular septal thickness
LVEF: Left ventricular ejection fraction
NORFOLK QoL-DN: Norfolk quality of life questionnaire for diabetic neuropathy
NIS: Neuropathy impairment score
NS: Not significant
NT-proBNP: N-terminal prohormone of brain natriuretic peptide
QoL: Quality of life
QRS: Q, R and S waves
QT: Q–T interval
PN: Polyneuropathy
RBP4: Retinol-binding protein 4
R–R interval: The time elapsed between two successive R-waves
SD: Standard deviation
THAOS: Transthyretin amyloidosis outcome survey
TTR: Transthyretin
