# Supplementary material for: A description of variant transthyretin amyloidosis (ATTRv) stage 1 patients and asymptomatic carriers in Spain: the EMPATIa study
Source: Orphanet J Rare Dis. 2024 Sep 6;19:323. doi: 10.1186/s13023-024-03304-9 (PMC11378489; doi:10.1186/s13023-024-03304-9)
Supplement: Supplementary file 1 — Supplementary Material 1. [file 13023_2024_3304_MOESM1_ESM.pdf]

Supplementary Figure S1

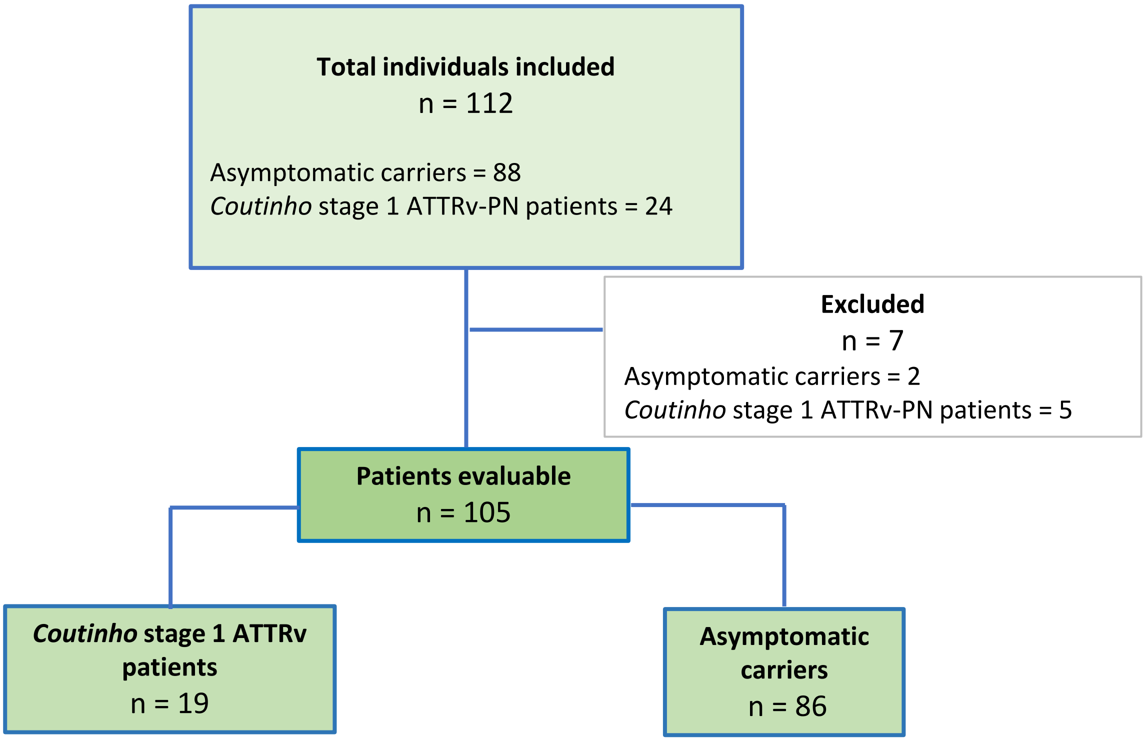

Supplementary Figure S1. Flow chart of study patient recruitment. Two asymptomatic carriers met exclusion criteria 2 (diabetes *mellitus*); four patients did not meet inclusion criteria 3 (diagnosed in the last 12 months), and one patient had both conditions (inclusion criteria 3 and exclusion criteria 2).
